# Project Score database: a resource for investigating cancer cell dependencies and prioritizing therapeutic targets

**DOI:** 10.1093/nar/gkaa882

**Published:** 2020-10-17

**Authors:** Lisa Dwane, Fiona M Behan, Emanuel Gonçalves, Howard Lightfoot, Wanjuan Yang, Dieudonne van der Meer, Rebecca Shepherd, Miguel Pignatelli, Francesco Iorio, Mathew J Garnett

**Affiliations:** Wellcome Sanger Institute, Hinxton, Cambridge CB10 1SA, UK; Open Targets, Wellcome Genome Campus, Hinxton, Cambridge CB10 1SD, UK; Wellcome Sanger Institute, Hinxton, Cambridge CB10 1SA, UK; Open Targets, Wellcome Genome Campus, Hinxton, Cambridge CB10 1SD, UK; Wellcome Sanger Institute, Hinxton, Cambridge CB10 1SA, UK; Wellcome Sanger Institute, Hinxton, Cambridge CB10 1SA, UK; Wellcome Sanger Institute, Hinxton, Cambridge CB10 1SA, UK; Wellcome Sanger Institute, Hinxton, Cambridge CB10 1SA, UK; Wellcome Sanger Institute, Hinxton, Cambridge CB10 1SA, UK; Wellcome Sanger Institute, Hinxton, Cambridge CB10 1SA, UK; Wellcome Sanger Institute, Hinxton, Cambridge CB10 1SA, UK; Open Targets, Wellcome Genome Campus, Hinxton, Cambridge CB10 1SD, UK; Human Technopole, 20157 Milano, Italy; Wellcome Sanger Institute, Hinxton, Cambridge CB10 1SA, UK; Open Targets, Wellcome Genome Campus, Hinxton, Cambridge CB10 1SD, UK

## Abstract

CRISPR genetic screens in cancer cell models are a powerful tool to elucidate oncogenic mechanisms and to identify promising therapeutic targets. The Project Score database (https://score.depmap.sanger.ac.uk/) uses genome-wide CRISPR–Cas9 dropout screening data in hundreds of highly annotated cancer cell models to identify genes required for cell fitness and prioritize novel oncology targets. The Project Score database currently allows users to investigate the fitness effect of 18 009 genes tested across 323 cancer cell models. Through interactive interfaces, users can investigate data by selecting a specific gene, cancer cell model or tissue type, as well as browsing all gene fitness scores. Additionally, users can identify and rank candidate drug targets based on an established oncology target prioritization pipeline, incorporating genetic biomarkers and clinical datasets for each target, and including suitability for drug development based on pharmaceutical tractability. Data are freely available and downloadable. To enhance analyses, links to other key resources including Open Targets, COSMIC, the Cell Model Passports, UniProt and the Genomics of Drug Sensitivity in Cancer are provided. The Project Score database is a valuable new tool for investigating genetic dependencies in cancer cells and the identification of candidate oncology targets.

## INTRODUCTION

The investigation of genetic dependencies in cancer cells is central to our understanding of oncogenic mechanisms and the discovery of therapeutic targets. Personalized medicine is revolutionizing the treatment of cancer patients, not only through the reduction of toxic and long-lasting side effects, but also by improving quality of life and overall survival rates. For example, targeting HER2 with trastuzumab in breast cancer (1), mutant BRAF with vemurafenib in melanoma (2) and EGFR with cetuximab in colorectal cancer (3) has transformed patient outcomes since their introduction into the clinic. However, much more needs to be done to realize the potential of cancer precision medicine. The development of new promising therapeutic targets has slowed down in recent years and many therapies fail due to the lack of efficacy in clinical trials (4,5). Furthermore, many patients have cancers with a molecular context that is not addressed in the clinic and lack personalized, targeted therapies. For these reasons, there is an urgent need to identify new and promising targets that can be successfully modulated for therapeutic benefit. A promising drug target should be selectively required for disease, tractable for drug development (so-called druggable targets) and, importantly, have an associated biomarker to select patients and monitor therapeutic efficacy (6). Targets with a genetic linkage to disease are more than twice as likely to progress through drug discovery pipelines and clinical trials to ultimately succeed in the clinic (7,8).

CRISPR–Cas9 whole-genome dropout screens are a powerful tool for the study of gene function and, when combined with downstream data integration, can identify promising therapeutic targets (9,10). High-throughput CRISPR–Cas9 genetic screens allow us to investigate the effect of gene knockout on cellular fitness (resulting in a cytostatic or cytotoxic effect) at a genome-wide scale across many different cancer types. Hundreds of genes are required for cell fitness and this varies by tissue type and molecular context, making it challenging to nominate the most promising candidate targets. To address this, we developed a computational framework to assign fitness genes a *target priority score* that integrates cellular fitness data with genomic biomarkers and target tractability for drug development to prioritize targets at both a pan-cancer and a cancer type-specific level (9). The target priority score can be used to guide selection of promising candidate therapeutic targets.

Here, we describe the Project Score database, which enables investigation of fitness effects and target priority scores for a gene, cell line model or cancer type of interest. Project Score is home to exclusive, freely available and downloadable datasets that will continue to increase in size as we generate more data. The database allows user-friendly, customizable and comprehensive navigation of datasets, with graphical summaries and interactive features. The website has been designed to allow for querying of specific biological hypotheses, or to browse data to generate new insights. The Project Score database is part of the Cancer Dependency Map (depmap.sanger.ac.uk) at the Wellcome Sanger Institute, which aims to identify all cancer cell dependencies to support precision cancer medicine, and linked with the Open Targets initiative (opentargets.org) (11) to facilitate new drug target identification.

## PROJECT SCORE DATA CONTENT

**Figure 1.**
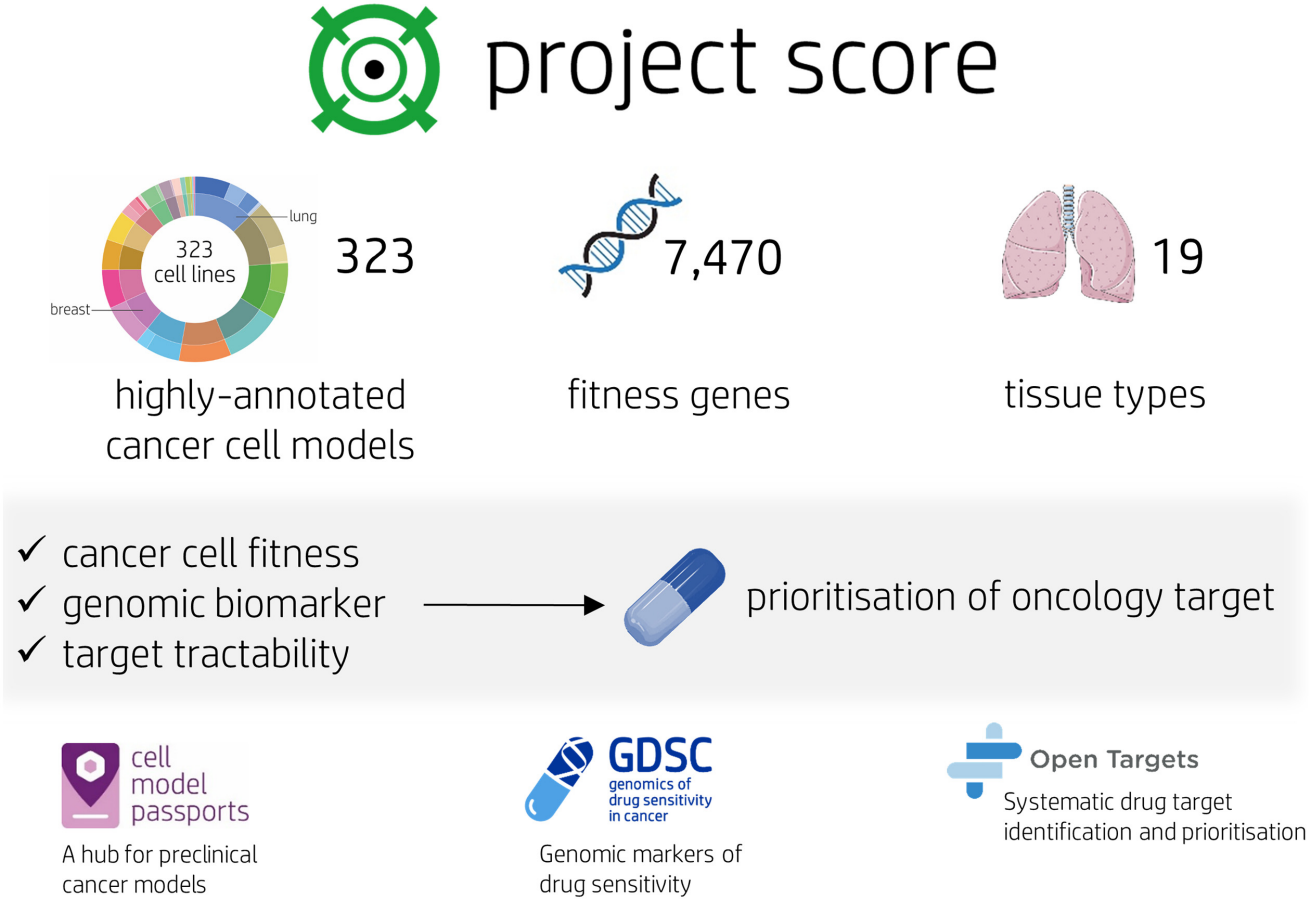
Project Score database overview: Part of the Cancer Dependency Map at Sanger, Project Score currently hosts genome-wide CRISPR–Cas9 knockout data for 323 cancer cell lines, identifying over 7000 fitness genes in 19 different tissue types. Gene fitness data are combined with genomic biomarker and tractability information to prioritize novel therapeutic targets. Further information on genes, drugs and targets can be explored via direct links to the Cell Model Passports, GDSC and Open Targets databases.

The current version of Project Score (version 2.0, released August 2020) provides access to whole-genome CRISPR–Cas9 gene knockout screens generated at the Wellcome Sanger Institute targeting 18 009 genes in 323 cancer cell lines from 19 different tissue types (Figure 1) (9). All cell line models have been extensively characterized, with genomic datasets including whole-exome DNA sequencing and RNA sequencing data freely available via direct links to the Cell Model Passports database (12). Similarly, cell line drug sensitivity data from pharmacological screens using full dose–response curves to over 500 anticancer compounds are available from the Genomics of Drug Sensitivity in Cancer (GDSC) database (13).

### Gene fitness scores

The Project Score database allows for the interrogation of 7470 fitness genes identified to be essential in at least one cell line, across multiple different cancer and tissue types. Gene fitness calculations are corrected for copy number bias using CRISPRcleanR (14). Cell line-specific gene fitness effects are provided as both log-fold change values, calculated as the difference between the average representation of target sgRNA 14 days post-transfection versus the plasmid library, and gene fitness scores, which are Bayesian factors from a statistical analysis using BAGEL (15). A gene fitness score of <0 indicates that a gene has a statistically significant effect on cell fitness.

The database also identifies cancer type-specific and pan-cancer core fitness genes. Core fitness genes are required for the fitness of the majority of cell lines within a cancer type or when considering all cancer types together in a pan-cancer analysis. A statistical method called the adaptive daisy model was used to determine the minimum number of dependent cell lines that are required for a gene to be classified as core fitness. Using this method, 553 pan-cancer core fitness genes were identified, including 132 genes never previously identified as human core essential genes (9).

### Target priority scores

To facilitate the identification of candidate drug targets, fitness genes are assigned a cancer type-specific and pan-cancer target priority score. Ranging from 0 to 100, the target priority score integrates multiple lines of evidence to prioritize a target. The majority of the priority score (70%) was derived from CRISPR–Cas9 experimental evidence. For each cell line, this cumulatively takes into consideration the fitness effect (BAGEL fitness score), the significance of this effect [MAGeCK *P*-value (16), based on targeting guide fold change], whether the gene is upregulated or mutated, and whether the gene belongs to a pathway that is statistically enriched for fitness genes. The remaining 30% of the target priority score was based on evidence of a biomarker associated with a dependency on the target, and takes into account the frequency at which the target is somatically altered in patient tumours (genomic biomarker prevalence). The strength of the associated genomic biomarker ranges from class A to class C (strongest to weakest) and is based on statistical significance of the target association and effect size. Core fitness genes have by default an assigned priority score of 0 (or null), as these targets have an increased likelihood of non-selective toxic effects in tissues. Using this pipeline, hundreds of genes and their protein products have been prioritized as potential therapeutic targets. Both cancer type-specific and pan-cancer priority scores are displayed on each gene’s web page, if available. This is accompanied by information on the presence of an associated genomic marker and fitness scores. Full documentation on how the target priority scores are generated, analysed and interpreted is available from the home page under ‘Documentation’. All target priority scores are available to download in tabular format from the ‘Downloads’ tab on the home page.

## DATA ANALYSIS AND VISUALIZATION

The Project Score database uses intuitive, dynamic and interactive interfaces to support data analysis and enhance the user experience. Interfaces are compatible with tablets and phones. Users can search by specific genes, cell lines or cancer type of interest from a search bar on the home page. Alternatively, all data can be explored in an unbiased fashion by selecting ‘fitness’ or ‘target priority’ scores under ‘explore data’ from the home page.

### Explore data: target priority and fitness scores

**Figure 2.**
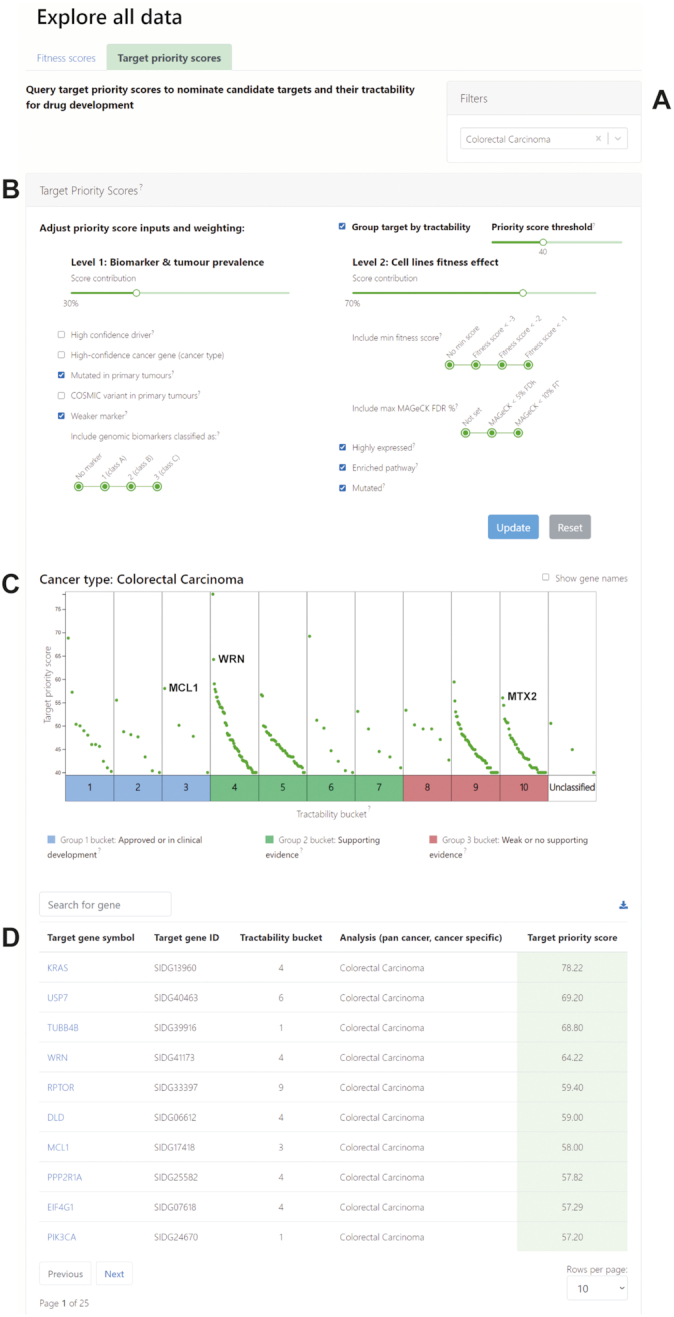
Target priority scores view. Target priority scores can be accessed and explored from the Project Score home page. Scores can be filtered by (**A**) cancer type and further refined by (**B**) biomarker and tumour prevalence, and fitness effect. (**C**) Target priority scores are represented by a green data point and are interactively presented in a colour-coded plot, grouping targets into tractability buckets. Selected examples of priority targets are labelled. (**D**) Priority scores in tabular format.

To browse target priority scores, ‘explore target priority scores’ can be selected from the home page. Target priority scores per cancer type or pan-cancer can be queried and filtered (Figure 2A). Default biomarker and fitness filters are displayed (Figure 2B), and these can be adjusted by users to customize their query. The weight between each level of prioritization (biomarker or fitness) can be adjusted, as well as the significance of genomic marker strength and fitness scores. All filter parameters are paired with a tool tip (represented by ‘?’) for further information and definitions.

Users can explore the tractability of the targets from the plot (Figure 2C), that is the existence of supporting evidence that we can identify a drug or compound that interacts with the target to elicit a desired biological effect, such as inhibition of activity. Each target is placed into one of 10 tractability buckets (Figure 2C; *x*-axis) based on tractability assessment for the development of small molecules and antibodies, with bucket 1 containing the most tractable targets and bucket 10 the least tractable (11). Tractability buckets are colour coded in the plot for clear identification. Buckets 1–3 (blue) contain targets of approved anticancer drugs or compounds in clinical or preclinical development; buckets 4–7 (green) contain targets without drugs in clinical development but that have evidence to support target tractability; and buckets 8–10 (red) contain targets with weak or no supporting information of target tractability. This information can be easily obtained from the tractability plot tool tips. Users can zoom into each bucket to more easily identify targets, and can toggle on and off gene name labels and grouping of targets by tractability. Hovering the cursor over a target provides the target name and priority score. Target priority scores can also be viewed in tabular format (Figure 2D) and range from highest to lowest scores.

In the example shown (Figure 2), candidate targets in colorectal cancer models are presented and grouped by tractability. Notably, this includes targets such as the BCL-2 family protein MCL1 involved in apoptosis that is the target of existing drugs in clinical development (tractability bucket 3), novel candidate targets such as WRN that have evidence for tractability (bucket 4), and MTX2 that is involved in mitochondrial function but has no evidence supporting tractability (bucket 10). Selecting a target gene links to the gene page for further information.

In addition to target priority scores, cellular fitness data for all genes can be accessed from the home page and filtered per cancer type. Gene fitness effects in each cell line are displayed in tabular format as both ‘corrected log-fold change’ and ‘loss of fitness score’. Statistically significant values are highlighted in green and labelled in the table. Pan-cancer core fitness genes are highlighted in red and can be excluded by checking a box in the filter panel. Users can rank scores from lowest to highest and vice versa, as well as search for particular genes of interest.

In summary, the ‘explore data’ pages enable scientists using default or user-specified criteria to investigate the fitness effect of thousands of genes across hundreds of cancer cell models and readily identify candidate oncology drug targets. In the following sections, we demonstrate how the Project Score database can be used for the detailed investigation of specific targets and cell models, using *WRN* helicase and the cell line RKO as representative examples.

### Gene view

**Figure 3.**
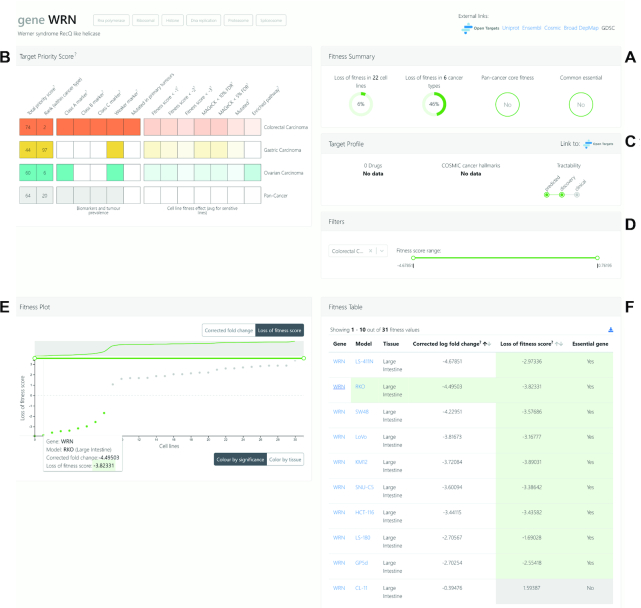
Querying the Project Score database by gene. (**A**) Fitness information on WRN in colorectal cancer is summarized in the ‘Fitness Summary’ panel, where users can identify the number of cell lines and cancer types that WRN has a fitness effect. (**B**) Target priority scores can be explored by cancer type or pan-cancer. (**C**) Information on the tractability of WRN can be viewed in the ‘Target Profile’ panel, with direct links to the Open Targets portal. (**D**) Information can be filtered per cancer type and users can explore fitness scores in each cell line via the interactive (**E**) waterfall plot and (**F**) fitness table. Data for the cell line RKO are highlighted in panel (F).

Specific genes can be queried from the search bar on the home page or accessed from the aforementioned ‘explore data’ pages. If a gene does not have a fitness effect in any cell lines, it is identified as ‘never essential’ in the search bar. When a gene is selected, the gene view page is loaded with access to a range of data that can be explored and interpreted through graphical representations with interactive features (Figure 3). The summary of fitness information on the gene of interest (in this case, *WRN* helicase) is immediately available, where the user can see how many cell lines and cancer types the gene results in a loss of fitness effect, and if the gene is a pan-cancer core fitness or common essential gene (Figure 3A). The graphical ‘Target Priority Score’ panel shows that *WRN* is a priority target pan-cancer, and also a cancer type-specific priority target in colorectal, gastric and ovarian cancer cells (Figure 3B). The information available within the panel includes colour-coded total priority scores and where the score ranks within each cancer type, the strength of the associated genomic marker (classes A–C, or weaker marker) and significance of the fitness score determined using MAGeCK and BAGEL.

The gene view page also includes a ‘Target Profile’ panel where key information about a target gene directly sourced from the Open Targets portal can be accessed (Figure 3C). The number of available compounds targeted to the gene’s protein product can be found here, along with the number of compounds in each phase of clinical trials when hovering the cursor over. Information on whether the gene promotes or suppresses a COSMIC cancer hallmark is presented, as well as information on target tractability. In the case of WRN, it is not associated with a COSMIC cancer hallmark, and there are no existing drugs in clinical development but there is precedence for tractability. A direct link to the Open Targets portal is provided for easy and quick access to extensive further information on the respective gene’s target profile page.

The fitness plot and associated table enable users to investigate the differential fitness effect of a gene knockout across cell lines (Figure 3D–F). A specific cancer type or pan-cancer analysis can be selected from the filter panel. Here, the fitness score range is also adjustable if a more stringent selection is desired. Changing these parameters immediately adjusts the interactive waterfall fitness plot and the fitness table. The fitness plot, which plots ‘cell lines’ (*x*-axis) against ‘corrected fold change’ or ‘loss of fitness score’ (*y*-axis), highlights cell lines with a significant loss of fitness effect in green (Figure 3E). The plot can also be toggled to be colour coded by tissue types. Hovering the cursor over each point on the plot interactively reveals the name of the cell line with relevant fitness information. The fitness table to the right of the graph displays information from the plot in tabular format (Figure 3F), and can be downloaded.

Besides the curated data for the database, several relevant external links are available from the gene view page. More information on a gene of interest can be found via direct links to UniProt, Ensembl, COSMIC, the Broad Institute’s DepMap portal and GDSC, at the top of the web page.

### Cell line model view

**Figure 4.**
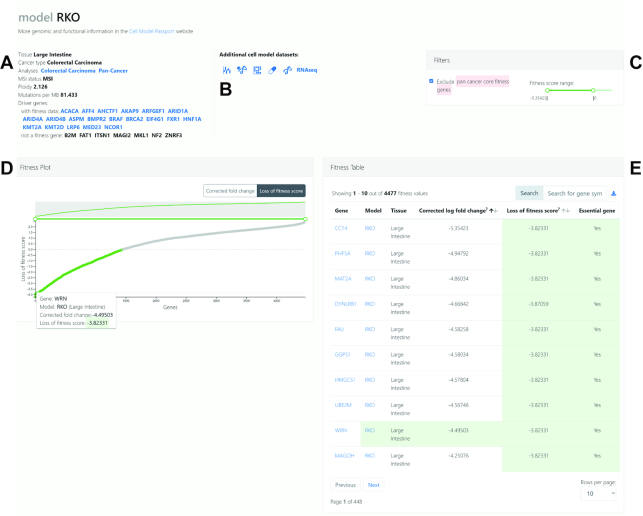
Querying the Project Score database by cell model. (**A**) Information on the colorectal cancer cell line RKO is summarized at the top of the page, with (**B**) direct links to relevant datasets on the Cell Model Passports database. (**C**) The range of fitness scores can be adjusted, and users can choose to exclude pan-cancer core fitness genes in the filter panel. (**D**, **E**) Gene fitness effects can be investigated in the interactive fitness plot and table.

It is also possible to investigate the fitness effects of all genes in each of the cell models. The cell model of interest can be queried from the search bar on the home page, selected from the gene view or from the ‘explore data’ pages. In the example shown (Figure 4), the colorectal cancer cell line model RKO has been selected from the fitness table on the *WRN* gene view page. Here, more information on the cell line model is presented, including microsatellite (MSI) status, mutations per megabase and cancer driver genes present in the model (Figure 4A). Available genomic and pharmacological datasets for the cell line are highlighted by visual icons with direct links to the Cell Model Passports database (Figure 4B).

The filter panel allows for adjustment of the fitness score range, as well as the default option to exclude pan-cancer core fitness genes (Figure 4C). The fitness plot is a similar layout to the gene view page, with ‘genes’ (*x*-axis) plotted against ‘corrected fold change’ or ‘loss of fitness score’ (*y*-axis) (Figure 4D). Significant loss of fitness genes are highlighted in green. The fitness table summarizes information from the plot and allows for navigation to fitness gene pages (Figure 4E), and can be downloaded in tabular format. As expected for RKO cells, *WRN* is among the top 10 fitness genes with the largest log-fold change effect size.

## DATA ACCESS

### Downloads

The downloads tab can be accessed from the website header. Here, raw and processed data files generated for Project Score can be downloaded. They include raw sgRNA read count files, copy number bias corrected fold change values (corrected using CRISPRcleanR) (14) and a fitness/non-fitness binary matrix data file for all cell lines used in the study. The same data downloads are available for CRISPR–Cas9 screens performed at the Broad Institute and analysed through the Project Score pipeline. Further to this, all target priority scores are available to download in bulk, tabular format from the downloads tab.

### API

The Project Score data are available from a RESTful API developed in Python 3.6 using the Flask framework (v0.12) and SQLAlchemy (v1.2). Requests can be made to the API at https://api.cellmodelpassports.sanger.ac.uk as previously described (12). Requests and responses follow the JSONAPI v1.0 standard (jsonapi.org). API documentation can be found at https://depmap.sanger.ac.uk/documentation/api.

Score data are available at the endpoint /datasets/crispr_ko. Further endpoints allow users to select results for particular cell models or genes using the corresponding Cell Model Passport identifiers, e.g. /models/SIDM00136/datasets/crispr_ko and /genes/SIDG01214/datasets/crispr_ko. Alternative identifiers can also be used, e.g. models/COSMIC_ID/905939/datasets/crispr_ko. The API can be searched to check whether Score data exist for a particular model or gene, e.g. /score_search/HT29. Finally, priority scores can be returned per cancer type analysis: /analyses/<analysis_id>/priority_scores, where the analysis id is, for example, 4 for colorectal carcinoma and 15 for pan-cancer.

## DOCUMENTATION

To support users, clear and concise documentation is provided, including a description of the experimental methods used for CRISPR–Cas9 screens and a detailed description of the gene fitness metrics. Details of the target prioritization score and target tractability assessment are also provided, as are links to key references and supporting information. The documentation also includes a glossary of commonly used terms and we have included extensive tool tips (represented with a ‘?’) throughout the database to provide information in a convenient fashion. In addition, documentation on the cell lines and genomic datasets used in the Project Score database are available from the Cancer Dependency Map at Sanger documentation portal (https://depmap.sanger.ac.uk/documentation/).

## DISCUSSION AND FUTURE WORK

Project Score is a new user-friendly database with unique functionality that enables scientists to explore genetic dependencies and potential therapeutic targets in cancer. The database is of interest to a wide range of basic and translational scientists, both bench and computer scientists alike, from academia and industry. Project Score is part of the Cancer Dependency Map at Sanger, which aims to identify all genetic dependencies in cancer to inform precision cancer medicine. This incorporates related complementary websites, including the Cell Model Passports (12), a database for preclinical cancer models, and GDSC (13), a resource of cell line drug response data and genomic markers of sensitivity. Project Score is also part of the Open Targets ecosystem of tools and websites and it is linked with the Open Targets portal, a freely available tool for the prioritization of drug targets in oncology and other diseases (11). Users can navigate to the Open Targets portal to obtain extensive information on their gene of interest, including available compounds and their clinical trial stages, target tractability, associated cancer hallmarks and biomarkers, pathway information, tissue expression levels and scientific literature based on text mining. Thus, the Project Score database is a valuable new tool linked within a rich ecosystem of complementary tools that collectively support cancer drug discovery.

Genome-wide CRISPR–Cas9 screening has emerged as a powerful tool to study cancer genomes and oncogenic phenotypes. This is illustrated by the emergence of CRISPR–Cas9 screening databases, such as GenomeCRISPR (17), used for the comparison of hits in screening experiments, PICKLES (18) and the Broad Institute’s own Cancer Dependency Map portal, used to explore gene essentiality and markers of gene dependencies. At the time of writing, however, Project Score is the only database that uses CRISPR–Cas9 experimental data for oncology drug discovery and includes a target priority score for each fitness gene, allowing the user to interpret the suitability of their target of interest. Moreover, the website integrates rich information on all fitness genes and cell lines from external sources in an accessible and intuitive manner.

The Project Score database will continue to grow and evolve as more data become available. Specifically, additional CRISPR–Cas9 screening datasets in cell lines are publicly available and, together with additional datasets generated at the Sanger Institute, these will be analysed through a consistent pipeline and integrated into the database in early 2021 (19,20). Additional datasets from CRISPR–Cas9 screens in patient-derived 3D organoid cultures and multi-gene CRISPR perturbations to study combinations of targets will be incorporated as they become available. Additional functionality will also be added, including an enhanced priority score pipeline and network-based visualizations of gene fitness effects and targets to improve interpretation and expand the repertoire of druggable targets. In conclusion, the Project Score database is a new user-friendly ever-growing resource that facilitates the discovery of candidate drug targets and a resource to catalyse precision cancer medicine.

## DATA AVAILABILITY

All data are available from the Project Score database.

## Notes

Present address: Fiona M. Behan, GlaxoSmithKline, Stevenage SG1 2NY, UK.
